# Total Vitamin D Assay Comparison of the Roche Diagnostics “Vitamin D Total” Electrochemiluminescence Protein Binding Assay with the Chromsystems HPLC Method in a Population with both D2 and D3 forms of Vitamin D

**DOI:** 10.3390/nu5030971

**Published:** 2013-03-22

**Authors:** Laila Abdel-Wareth, Afrozul Haq, Andrew Turner, Shoukat Khan, Arwa Salem, Faten Mustafa, Nafiz Hussein, Fasila Pallinalakam, Louisa Grundy, Gemma Patras, Jaishen Rajah

**Affiliations:** 1 Pathology & Laboratory Medicine Institute, Sheikh Khalifa Medical City, Abu Dhabi 51900, UAE; E-Mails: haq2000@gmail.com (A.H.); aturner@skmc.ae (A.T.); asalem@skmc.ae (A.S.); fmostafa@skmc.ae (F.M.); nnimer@skmc.ae (N.H.); fpallinalakam@skmc.ae (F.P.); lgrundy@skmc.ae (L.G.); gpatras@skmc.ae (G.P.); 2 Pathology & Laboratory Medicine Department, Military Hospital, Riyadh 11159, Kingdom of Saudi Arabia; E-Mail: sakhan@rmh.med.sa; 3 Pediatric Institute, Sheikh Khalifa Medical City, Abu Dhabi 51900, UAE; E-Mail: jrajah@skmc.ae

**Keywords:** 25-hydroxyvitamin D, method evaluation, electrochemiluminescence, high performance liquid chromatography

## Abstract

This study compared two methods of assaying the 25-hydroxylated metabolites of cholecalciferol (vitamin D3) and ergocalciferol (vitamin D2). A fully automated electrochemiluminescence assay from Roche Diagnostics and an HPLC based method from Chromsystems were used to measure vitamin D levels in surplus sera from 96 individuals, where the majority has the D2 form of the vitamin. Deming regression, concordance rate, correlation and Altman Bland agreement were performed. Seventy two subjects (75%) had a D2 concentration >10 nmol/L while the remaining twenty four subjects had vitamin D2 concentration of less than 10 nmol/L by HPLC. Overall, the Roche Diagnostics method showed a negative bias of −2.59 ± 4.11 nmol/L on the e602 as compared to the HPLC with a concordance rate of 84%. The concordance rate was 91% in samples with D2 of less than 10 nmol/L and 82% in those with D2 concentration >10 nmol/L. The overall correlation had an *r* value of 0.77. The *r* value was higher in samples with D2 levels of less than 10 nmol/L, *r* = 0.96, as compared to those with D2 values of greater than 10 nmol/L, *r* = 0.74. The observed bias had little impact on clinical decision and therefore is clinically acceptable.

## 1. Introduction

The last decade has witnessed a dramatic increase in both clinical and public awareness of the health implications associated with vitamin D status [1]. Consequently, clinical laboratories are receiving an increasing number of requests to measure vitamin D levels, which has led to the need for highly automated assays. A recent publication evaluated the performance of six routine 25-Hydroxyvitamin D assays in relation to variation in vitamin D binding protein concentration [2]. The majority of the study population had the D3 form of the vitamin (cholecalciferol). In contrast, our study evaluates the performance of one of those assays, the Roche Diagnostics “Vitamin D total” electrochemiluminescence protein binding assay, in a population where the majority have the D2 form of the vitamin (ergocalciferol). The technical performance of the Roche assay was evaluated by comparison to the Chromsystems HPLC-UV assay and the clinical performance was assessed in terms of classification of subjects as sufficient or deficient for the vitamin.

## 2. Material and Methods

### 2.1. Samples

Left over sera obtained from specimens analyzed at the SKMC Pathology & Laboratory Medicine Institute (*n* = 96) were used in this study. The use of this material was approved by the institutional ethics review board and is in accordance with the general consent signed by all patients prior to treatment at SKMC. Serum aliquots were given unique sample numbers, to conceal the identity of the patient from the staff performing the study. These aliquots were stored at 2–8 °C and analyzed within two days. 

### 2.2. Analytical Platforms

#### 2.2.1. Chromsystems HPLC Assay

The Chromsystems reagent kit used on the Waters HPLC 2695 allows the main metabolites of vitamin D3 and D2 to be determined in a simultaneous chromatographic manner by using a fully validated, modified high-performance liquid chromatography (HPLC) method [3]. The Waters HPLC 2695 analyzer uses a simple isocratic HPLC system, with a HPLC pump, injector and a UV detector. In summary, protein is precipitated, and through selective solid phase extraction, interfering components are removed and the analytes are concentrated. A stable vitamin D derivative is used as an internal standard in order to allow for accurate quantification. The chromatographic separation takes approximately 12 min (Chromsystems Instruments & Chemicals GmbH, Heimburgstrasse, Munich, Germany) [3]. The assay has within run imprecision of 3.0% and total (between days) imprecision of 4.6%.

#### 2.2.2. Roche Diagnostics Vitamin D Total Assay

The Roche Diagnostics Vitamin D total assay is a competitive electrochemiluminescence protein binding assay intended for the quantitative determination of total 25-OH vitamin D in human serum and plasma. The assay employs a vitamin D binding protein (VDBP) as capture protein, which binds to both 25-OH D3 and 25-OH D2 (Roche Diagnostics, Mannheim , Germany) [4].

The assay utilizes a 3-step incubation process, which has a duration of 27 minutes. In step 1, the sample is incubated with pretreatment reagent, which releases bound 25-OH vitamin D from the VDBP. In step 2, the pretreated sample is incubated with ruthenium labeled VDBP creating a complex between the 25-OH vitamin D and the ruthenylated VDBP. The third incubation step sees the addition of streptavidin-coated microparticles and 25-OH vitamin D labeled with biotin. The free sites of the ruthenium labeled VDBP become occupied, forming a complex consisting of the ruthenium labeled vitamin D binding protein and the biotinylated 25-OH vitamin D. The entire complex becomes bound to the solid phase via interaction of biotin and streptavidin.

Between day precision was CV = 4.9% and 1.9% at mean concentrations of 43.3 and 105 nmol/L respectively using quality control material provides by Roche Diagnostics.

Both assays were validated in our laboratory following “Clinical Laboratory Standards Institute” (CLSI) protocols for validation of precision, linearity and accuracy. 

Reference ranges used in this study were based upon the recommendations of the American Society for Bone and Mineral Research, 28th Annual Meeting 2006 and the Canadian consensus conference on osteoporosis, 2006 [5,6] and were defined as follows: Deficiency: <25 nmol/L, Optimal/Sufficiency: 75–200 nmol/L, Insufficiency: 25–75 nmol/L and Toxicity: >250 nmol/L. This study also considered the latest recommendations published by the Institute of Medicine (IOM) for dietary reference intake for calcium and vitamin D. According to the latest IOM recommendations, 25(OH) D levels corresponding to a serum 25(OH) D status of at least 50 nmol/L indicates sufficiency [1].

#### 2.2.3. Statistical Analysis

All data points were included in the study. Results were classified into three groups; the entire population, those with vitamin D2 concentration of less than 10 nmol/L and those with vitamin D2 concentration greater than 10 nmol/L. Method comparison was performed by using Deming regression. Method agreement was analyzed by the mean difference method of Bland and Altman. Pearson correlation was also calculated for the three groups. In addition, linear regression for the difference between the Roche method and the HPLC method in relation to concentration of vitamin D2 and D3 was performed to determine the influence of increasing concentrations of each of the forms respectively. Analysis was performed using “Analyse-it” (The Tannery, 91 Kirkstall Road, Leeds, LS3 1HS, UK) and Microsoft Excel softwares (Thames Valley Park Reading, Berkshire, RG6 1WG, UK).

The concordance rate was calculated by classifying the results obtained on each platform as sufficient, insufficient or deficient using the criteria already established in our laboratory as stated earlier as well as those recently suggested by the Institute of Medicine (IOM) [1].

Secondary analysis was performed to determine the predicted bias as the data demonstrated a non-constant scatter. The data was classified into three groups (low, middle and high) in order of increasing values based on the HPLC results. Predicted bias was then calculated using partitioned residuals as described in CLSI guideline EP09-A2-IR Section 6.3. Polynomial regression analysis was performed to determine the polynomial fit equation for each group.

## 3. Results

### Samples

A total of 96 samples were analyzed. Seventy two samples had D2 concentrations greater than 10 nmol/L as detected by the HPLC, while in the remaining twenty four the concentration of D2 was less than 10 nmol/L. None of the samples analyzed were hemolyzed or lipemic. The mean, standard deviation, median and the range were; mean 65.22 and 62.12 nmol/L ± 30.38 and 29.54, median 60.90 and 57.8 nmol/L, range 16.3–180.9 and 7.5–175.0 nmol/L, for the Chomsystem HPLC and Roche Diagnostics Cobas e602 respectively.

**Figure 1:**
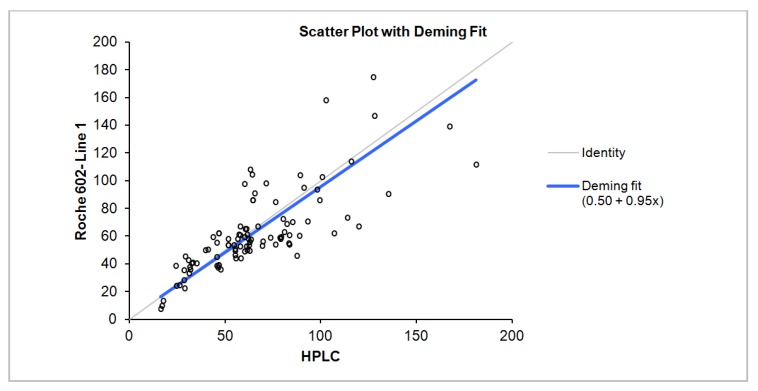
Deming regression of total 25(OH) D comparison: Chromsystems HPLC as the reference method *vs.* Roche Diagnostics Vitamin D total method on the Cobas e602.

The Roche Diagnostics method demonstrated a negative bias of −2.59 nmol/L (95% CI = −6.7–1.52) when compared to the HPLC method using the Bland-Altman analysis (Figure 1).

**Figure 2:**
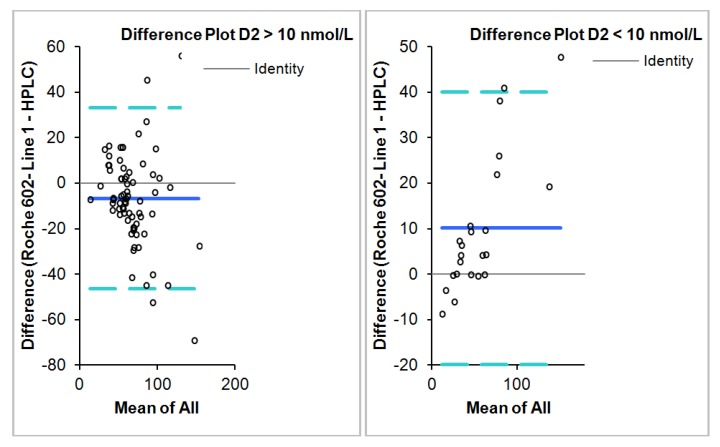
Bland-Altman plot showing means of paired difference between the HPLC method and the Roche Diagnostics Cobas e602 in samples with D2 greater than and less than 10 nmol/L respectively.

**Figure 3:**
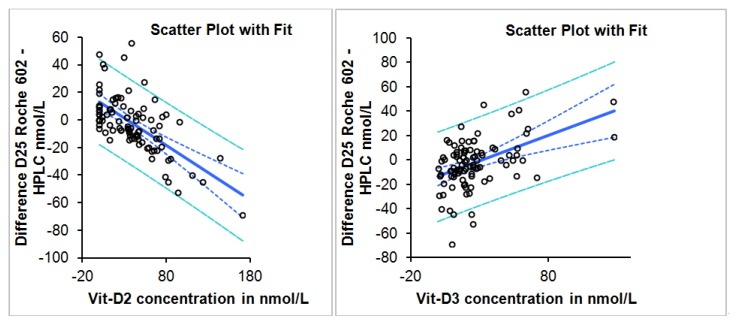
Linear regression analysis scatter plot of the difference in concentration between Roche Diagnostics total 25 (OH) and HPLC as a function of D2 and D3 concentrations.

Deming regression result, correlation, slope and intercept for all samples, samples with D2 concentrations less than and greater than 10 nmol/L is summarized in Table 1. The difference between the two methods is dependent on the concentration of D2 and D3 with negative bias observed more with increasing D2 concentrations and positive bias with increasing D3 concentration (Figure 2). Linear regression analysis scatter plot of the difference in concentration between Roche Diagnostics total 25 (OH) and HPLC as a function of D2 and D3 concentrations is shown in Figure 3.

**Table 1:** Person correlation, bias as calculated by Bland—Altman comparison, slope and intercept according to Deming regression for all samples, samples with D2 concentration less and more than 10 nmol/L as determined by HPLC.

Sample Group	*n*	Concentration Range in nmol/L	*r*	Bias	Slope	Intercept
All samples	96	16.30–180.9	0.77	−2.59	0.95	0.5
D2 concentration <10 nmol/L	24	16.30–127.80	0.96	10.14	1.43	−11.81
D2 concentration >10 nmol/L	72	16.90–180.90	0.74	−6.63	0.79	8.07

The concordance rate between the Cobas e602 and HPLC was 74% using the 75 nmol/L cutoff value as indication of vitamin D sufficiency. When applying the cutoff proposed by the IOM [1] the concordance rate increased to 84%. The concordance rate was 91% in samples with D2 of less than 10 nmol/L and 82% in those with D2 concentration >10 nmol/L. The majority of the disagreement was in the sufficient to insufficient range as the Roche method slightly underestimated at the higher concentrations (>50 nmol/L). There was no major discrepancy as to sufficiency *versus* deficiency noted. The detail of the sub classification is summarized in Table 2 for the two cutoffs respectively.

**Table 2:** Concordance of HPLC and Roche Diagnostics assays to 25 (OH) D based on 2 decision criteria.

Concordance Based on the 75 nmol/L Cutoff					Concordance Based on the 50 nmol/L Cutoff				
Roche	70–25 Cutoff	HPLC			Roche	50–30 Cutoff	HPLC		
		Sufficient	Insufficient	Dificient			Sufficient	Insufficient	Dificient
	Sufficient	13	7	0		Sufficient	60	6	0
	Insufficient	17	53	1		Insufficient	7	15	2
	Dificient	0	0	5		Dificient	0	0	6

The calculated predicted biases as absolute values and percentages at four medical decision limits (25 and 75 nmol/L) and (30 and 50 nmol/L) are summarized in Table 3.

**Table 3:** Calculated predicted biases at four medical decision cutoffs for low, middle and high groups.

Medical Decision Cutoff	Group 1	Group 2	Group 3
	(16.3–51.5 nmol/L)	(54.2–69.6 nmol/L)	(71.1–180.9 nmol/L)
	Predicted Bias (%)	Predicted Bias (%)	Predicted Bias (%)
25 nmol/L	2.6 (10.4%)	Not applicable for this group	Not applicable for this group
30 nmol/L	2.9 (9.6%)	Not applicable for this group	Not applicable for this group
50 nmol/L	Not applicable for this group	−4.0 (8%)	−1.57 (3%)
75 nmol/L	Not applicable for this group	11.27 (15%)	−7.7 (10.3%)

## 4. Discussion

Testing for vitamin D is required not only to screen for its deficiency, but also increasingly to adjust “therapeutic targets” and monitor efficacy of treatment. In addition to the skeletal effects, vitamin D may have a role in relation to diabetes, cancer and cardiovascular diseases [7,8,9,10,11,12,13]. Growing awareness of the clinical importance of vitamin D has resulted in clinical laboratories receiving a surge in requests for the assay. This has led to a need to migrate vitamin D testing from labor intensive methods, such as HPLC, to more highly automated testing platforms. Until recently the major challenge with vitamin D analysis has been the lack of standardization and the wide analytical variation between methods due to absence of reference standards [14]. The situation is further complicated by the discovery of C-3 epimers of vitamin D2 and D3 particularly in infants that might interfere with the accurate measurement and interpretation of vitamin D status in infants [15]. Recently, the National Institute of Standards and Technology (NIST) developed standard reference materials (SRMs) for 25(OH) D3/D2 in both human serum (SRM 972) and in solution (SRM 2972) [16]. This was supported by the recent introduction of reference measurement procedures using isotope—dilution liquid chromatography-tandem mass spectrometry [17].

The Roche Diagnostics Total Vitamin D kit has 80% cross reactivity to D2 and 100% cross reactivity to D3. This method was recently evaluated and its performance was deemed satisfactory in a population where D3 constituted the main form of total vitamin D [2]. In the United Arab Emirates, vitamin D deficiency is prevalent and the majority of the deficient population is receiving D2 supplementation [18]. In this study 75% of the participants had D2 levels >10 nmol/L. In this population, the Roche Diagnostics method had an overall negative bias, which was directly dependent on the increasing concentration of D2 as compared to the HPLC method. However, it was also observed that the method has positive bias, which was dependent on the concentration of D3 and therefore the overall negative bias was attenuated when both forms are present. More subjects were classified as “insufficient” by the Roche Diagnostics method. However, the clinical impact of the slight underestimation, one can argue, is of little clinical significance giving the wide range for the optimum level of vitamin D (50–150 nmol/L). The analytical quality goals for 25-vitamin D based on biological variation were a subject of a recent publication [19]. According to this publication, the desirable analytical bias goal is around 10% and 6% for the imprecision [20]. The calculated predicted bias using the was found to be within 10% when the IOM decision criteria of 30 and 50 nmol/L were applied and within 15% when the criteria of American Society for Bone and Mineral Research were applied. The method has an imprecision of less than 5%. We subscribe to the UK-based DEQAS vitamin D external quality assessment scheme. According to DEKAS 2012 review report, the Roche total vitamin D method had a mean % bias of less than 10% from all laboratory trimmed mean (ALTM) while the IDS RIA and IDS iSYS assays had a high positive bias which reached up to 21%. LC-MS method had positive bias according to this review of less than 10% while HPLC method had a positive bias slightly higher than 10% in one of the cycles [21]. Since both methods in this study used different standards, a clear cut 100% concordance on all samples might not be achievable. Adding to the lack of standardization are the inherent technical limitations of the protein binding assays relative to the direct chemical methods, the effect of vitamin D binding protein concentration of the results and the possible interferences from the C-3 epimers of D2 or D3 forms on the HPLC method [15].

## 5. Conclusions

In conclusion, Roche Diagnostics “Vitamin D total” assay performance in a population where the D2 component is negatively biased when compared to the HPLC Chromsystems method, and this is directly related the concentration of D2. The negative bias is less often observed when D3 is also present. The negative bias may impact classification of individuals receiving the test in terms of sufficiency or insufficiency for vitamin D and it should be taken in consideration when interpreting results of patients on D2 supplementation. The observed bias had little impact on clinical decision when the IOM criteria were applied and therefore is clinically acceptable.
